# Molecular characterization and phylogenetic study of peste des petits ruminants viruses from North central States of Nigeria

**DOI:** 10.1186/1746-6148-7-32

**Published:** 2011-07-04

**Authors:** Pam D Luka, Joseph Erume, Frank N Mwiine, Chrisostom Ayebazibwe, David Shamaki

**Affiliations:** 1 Department of Parasitology and Microbiology, School of Veterinary Medicine, Makerere University, Kampala, P. O. Box 7062, Kampala, Uganda; 2 Biochemistry and Applied Molecular Biology Division, National Veterinary Research Institute, PMB 1 Vom, Nigeria; 3 Department of Veterinary Medicine, School of Veterinary Medicine, Makerere University, Kampala, P. O. Box 7062, Kampala, Uganda; 4 Ministry of Agriculture, Animal Industry and Fisheries, P.O. Box 513, Entebbe, Uganda; 5 Directorate of Research, National Veterinary Research Institute, PMB 1 Vom, Nigeria

## Abstract

**Background:**

Peste des petits ruminants is an endemic disease of sheep and goats in Nigeria and vaccination has been the method of control but sporadic outbreaks have been reported. This study was carried out to characterize PPR viruses from outbreaks in 2007 and 2009 from Kaduna and Plateau States.

**Results:**

Of the 33 clinical samples analysed, 51.52% (n = 17) were positive for F protein gene primers (F1/F2). All the samples had a sequence similarity of 98-100% among them and 92-97% with the reference vaccine (Nig 75/1) strain. The deduced amino acid homology ranges between 96.3-99.7%. Phylogenetically all the Nigerian sequences cluster with Nig 75/1 and Nig 76/1 in lineage 1.

**Conclusions:**

PPR is still a problem in Kaduna and Plateau States of Nigeria. The strains involved were genetically closely related to the vaccine strain (Nig 75/1) used in the country. Based on this study, the continued outbreaks in the Country is not due to the efficacy of the vaccine. Therefore, to achieve effective control and possibly eradication of PPR in Nigeria, the current control strategies should be revisited.

## Background

Peste des petits ruminants disease (PPR), caused by PPR virus (PPRV), is a highly contagious disease of sheep and goats that has been widely reported in Sub Saharan Africa [1]. The disease has been characterized by fever, erosive-ulcerative stomatitis, fibrino-necrotic tracheitis, brochointestitial pneumonia and diarrhea [2,3]. Morbidity and mortality can be as high as 100% and 90%, respectively, depending on the endemic status of the disease in an area [2].

This disease was first described in West Africa in 1940s [4] and the first outbreak was describe in Nigeria in 1975 by Taylor and Abegunde [5]. The virus has since spread to other countries. PPR has been reported in Southern Asia, Near East, Arabian Peninsula and in recent years outbreaks have been reported in Turkey (2000), Tibet China (2007), Morocco (2008), Tanzania (2008) and Uganda (2007). Since the earlier reports on Nigeria in 1975, the disease has become endemic giving rise to economic loses to the rural poor who rely on these animals as a source of livelihood.

Earlier studies have suggested that PPR might have been around for quite some time in different countries but was wrongly diagnosed [6]. It has also been confused with rinderpest because of the clinical similarity [7]. Laboratory techniques that were used in diagnosis were virus neutralization test (VNT), agar gel immunodiffusion test (AGID), counter immuno-electrophoresis (CIEP) and virus isolation which has been time consuming and laborious [8]. Recently, molecular biology tools have made it possible to diagnose this disease rapidly and with great sensitivity compared to earlier test [9,10].

Etiological agent of PPR is a member of the family Paramyxoviridae and genus morbillivirus [11]. The viral genome is 15,948 nucleotides long [12] and contains six genes encoding six major polypeptides: nucleocapsid protein (N), phosphoprotein (P), matrix protein (M), fusion (F) protein, hemagglutinin (H), and large RNA-dependent polymerase protein. It is a linear, single stranded, non segmented, negative sense RNA virus [11,12]. Although PPRV has been known to occur as one strain or serotype [13], partial sequence analysis of the fusion protein gene, indicates occurrence of four lineages (1, 2, 3 and 4), of which three have been reported in Africa [13]. The fourth (4th) lineage is the only one that exists in the Indian sub continent but East African lineage 3 has been reported to coexist with the lineage 4. This phylogenetic analysis is helping in the epidemiological understanding of the spread of the disease among animal populations. In Nigeria there is continuing pockets of outbreaks of PPR in small ruminants necessitating studies to delineate the molecular details of the circulating field virus (es). The aim of the present study was to determine the phylogenetic relatedness of PPR viruses from outbreaks in the North central States of Nigeria.

## Results

### Detection of PPRV F gene

**Figure 1:**
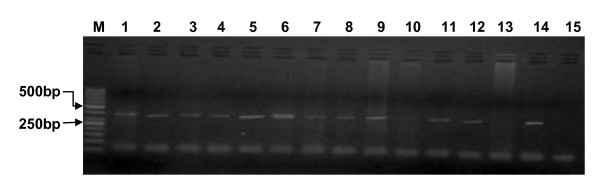
**PCR amplicon for F gene showing band size of 372 bp**. (from the left - right) First Lane M: molecular marker of 50 bp, Lane 1-13: samples and Lane 14, 15: positive and negative controls respectively.

Of the 33 clinical samples collected from sheep (*n *= 20) and goats (*n *= 13), 17 were positive for PPRV by F1/F2 primer amplification (Positivity rate = 51.52%). The remaining 16 samples were negative for PPRV. All the 7 samples collected from Kaduna were positive while out of the 26 samples from Plateau only 10 gave the target 372 bp amplicon (Figure 1. PCR amplicon for F gene showing band size of 372 bp). The reference vaccine strain (Nig 75/1) also yielded the 372 bp amplicon.

### Sequencing

The 372 bp region of the F gene of 17 PPRV positive samples were obtained. Of the ten (10) positive PCR products, nine (9) sequences were generated (7 from Plateau and 2 from Kaduna). One sample (VRD/348/09, from Plateau) did not yield any sequence. The remaining 7 positive samples were not sequenced. All the 9 sequences were submitted to the GenBank (Accession number: HQ317871 - HQ317879)

### Sequence similarity analysis

**Figure 2:**
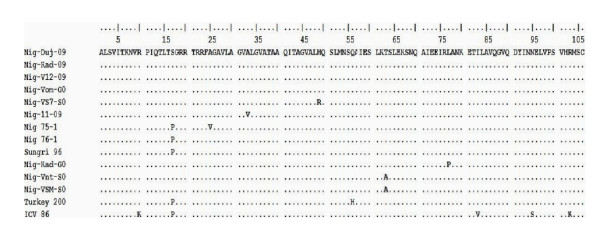
**Sequence alignment of 106 deduced amino acid of the 322 fragment of the F gene region of 11 Nigerian PPR viruses with that of India (Sungri), Turkey (Turkey 2000) and Cote d'Ivoire (ICV86) showing amino acid variations**.

A sequence comparison of the nine (9) 322 bp F gene sequence fragments showed 98-100% nucleotide homology. The two sequences from Kaduna (HQ317873 and HQ317874) showed 99% nucleotide homology while the seven sequences from Plateau showed 98-100% similarities. Two sequences (2) from Plateau (HQ317876 and HQ317879) were entirely identical in their nucleotide. Inter- State, one (1) sequence sample from Kaduna (HQ317873) and one (1) from Plateau (HQ317877) were also entirely identical among them. All the nine (9) field sequences also showed 93-95% nucleotide similarity with the vaccine. Identity levels of deduced Amino acid sequences of the 9 samples ranged from 96.3-99.7% **(**Figure 2. Alignment of deduced amino acid of Nigeria PPR and other sequences).

### Phylogenetic analysis

**Figure 3:**
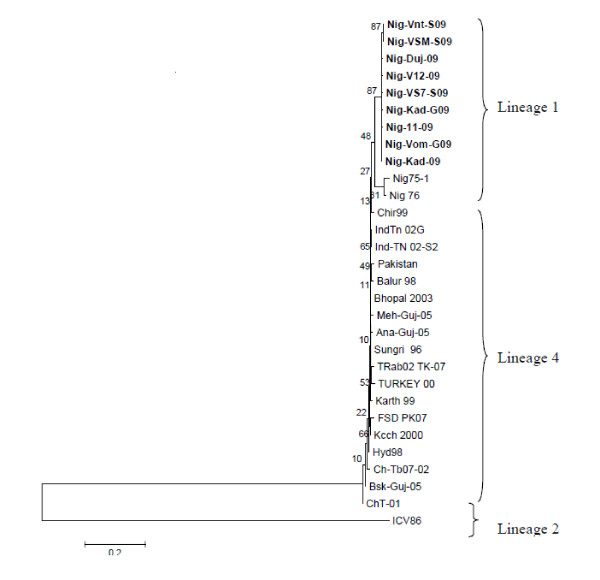
**Phylogenetic relationships between peste des petits ruminants virus (PPRV) detected from Nigeria samples in 2007/2009 with other sequences from the GenBank**. PPRV strains sequenced in this study are highlighted. Phylogenetic analyses were completed with MEGA 4 software that used a neighbour-joining algorithm.

The results of phylogenetic analysis of the sequences (n = 9) obtained from this study based on the 322 bp lineage specific sequences of F gene are shown in Figure 3. They were aligned with other available sequences from the GenBank, including sequences from Turkey, India, Pakistan and China. All the Nigerian sequences clustered into lineage 1 together with the vaccine strain Nig 75/1 and Nig 76/1 separate from the Turkey and Indian strain which belong to the lineage 4. None of the sequences clustered with Cote d'Ivoire which belongs to lineage 2. There were no sequences of lineage 3 in the GenBank to be used in generating the tree. From Plateau State, HQ317876 (Nig/Vnt/S09) and HQ317879 (Nig/VSM/S09) showed close relationship (Figure 3 Phylogenetic tree of Nigeria sequences and other sequences from the GenBank). These findings suggested that the Nigerian PPR viruses vary in the level of similarity but are homologous and similar to the vaccine strain isolated in 1975 (Nig 75/1) used in the country.

## Discussion

PPR is a very serious economic disease that has persisted in Nigeria for decades. One of the most important outbreaks occurred in 1975 and 1976 [5]. Since then the disease has continued with outbreaks occurring sporadically [14]. Due to the endemic situation in Nigeria, control has been mainly hinged on vaccination [15]. Outbreaks are detected clinically and confirmed either serologically or using molecular techniques. The F gene based RT-PCR developed by Forsyth and Barrett [9] has gained much popularity, though PCR tools based on other gene targets have become available [10]. The PPR specific primers F1 and F2 developed by aforementioned workers give an amplification of 372 bp between positions 777 to 1148 nucleotides of PPRV F gene. In the present study, PPRV was detected by F gene based RT-PCR in 17 (51.52%) of the 33 clinical samples tested, which confirmed PPRV in the two North central states. Previous survey by Obi and Ojeh [16] in Nigeria reported a positivity rate of 86.8% and 81.6% from tissue homogenate using dot ELISA and standard Indirect ELISA, respectively, from the Southern States of Nigeria. Our findings suggest that PPR is less prevalent in the Northern States (Kaduna and Plateau) as opposed to the Southern. Arguably, the differences in positivity rate may also be related to the detection method, sample size and the presence of other exacerbating diseases such as pasteurellosis, caprine contagions pleuropneumonia (CCPP), blue tongue (BTV), contagious ecthyma and foot and mouth disease (FMD) [17] which can occur concurrently. Nonetheless the PPR positivity rate we found was similar to that reported recently in an endemic region in India (50%) using RT-PCR [18].

Although PPR has been a problem in Nigeria for a long time, studies did not go as far as sequencing of the isolates apart from the full genomes sequence of Nig 75/1 (vaccine strain) and Nig 76/1 (field isolate) which were done in Pirbright [12,19]. Our study is the first to carry out sequencing and sequence analysis of F gene from PPR viruses involved in the recent disease outbreaks (2007 and 2009). A sequence comparison showed high level of homology (98-100%) of the circulating viruses suggesting that these viruses do not undergo rapid genetic changes in the F gene. Our data appear to be in agreement with other studies [18,20] who reported that the PPR virus is more prone to mutations on the N gene compared to the F gene. Between States, the PPR viruses were also highly homologous. This suggests criss-cross movement of infected small ruminants between States. Geographically, the two states share boundaries but also human related activities such as persons buying and transporting animals which may account for long distance spread of PPR. According to Ezeibe et al., [21] vaccinated animals can shed the virus from faeces for up to 12 weeks post vaccination. Arguably, naturally infected animals if moved from place to place or State to State can form a big source of infection to naive populations.

Approximately 35 years ago, PPR was reported in Nigeria from sheep and goats purchased from open markets at Vom and Potiskum [5]. Since then the tissue culture rinderpest vaccine (TCRV) was used to confer protection against PPR until recently when the homologous PPR vaccine was developed by Diallo [22] from the attenuation of Nig 75/1 PPRV strain. Interestingly, all the Nigerian strains from this study demonstrated 93-95% homology to the latter vaccine strain currently used for the control of the PPR in Nigeria. Our study revealed that the Nig 75/1 vaccine is indeed the suitable one for use in Nigeria. Based on our findings, the continued occurrence of outbreaks in Nigeria may not be attributed to the choice of vaccine but rather on other factors such as inadequacies related to the control strategy. Indeed Annatte et al., [23] have reported lapses in Veterinary extension services in relation to PPR control in Lagos State, the situation may not be different in other States.

A consensus Phylogenetic tree based on the lineage specific 322 bp of F gene sequence was constructed with the help of MEGA 4 program. In accordance with the previous studies [13-15,24], all the Nigerian strains including the vaccine strain Nig 75/1 and Nig 76/1 isolate clustered together. All the Asian and Turkish isolates, clustered together into a separate branch from the Nigerian isolates, and the branching was supported by bootstrap values of 500 (100%). None of the sequences clustered with the strain from Cote d'Ivoire which belongs to lineage 2. Since the sequence data for the African isolates belonging to lineage 3 were not available in the Genbank, they were not included in the Phylogenetic analysis. Thus, from the Phylogenetic tree (Figure 3), it was evident that all the isolates of Kaduna and Plateau clustered together with Nig 75/1 and Nig 76/1 isolates in lineage 1, reinforcing the findings that these isolates are homologous. Epidemiologically these data suggest that there has been no introduction of any new PPRV strain into Nigerian small ruminant populations.

## Conclusion

The data from this study have shown that PPR is a problem in Kaduna and Plateau States of Nigeria. The strains involved were genetically closely related to the vaccine strain (Nig 75/1) used in the country. Based on these data, the continued outbreaks in the Country is not due to the efficacy of the vaccine. Therefore, to achieve effective control and possibly eradication of PPR in Nigeria, the current control strategies should be revisited.

## Methods

### Clinical samples and the reference vaccine strain

A total of 33 (7 from Kaduna and 26 from Plateau State) clinically suspected PPR tissue samples (lymph node, spleen, liver, lungs and intestine) of sheep (*n *= 20) and goats (*n *= 13) from outbreaks were collected from Kaduna and Plateau States by field Veterinary Officers in 2007 and 2009, respectively. The samples were kept at -70°C at the Central Diagnostic Laboratory of the National Veterinary Research Institute (NVRI), Vom, Plateau State. PPR vaccine strain (Nig 75/1) from Pestvac^®^, Jordan was used as a reference strain virus in this study.

### Shipping of samples

Permission to use the samples (Additional File 1: Appendix 1) were obtained from the Executive Director NVRI and shipped into Uganda with an import permit (Additional File 2: Appendix 2) from the Commissioner Animal Health and Entomology, Ministry of Agriculture Animal Industry and Fisheries, Entebbe. Standard procedure was followed as required by the Office of International Epizootics (OIE) and International Air Transport Authority (IATA) to ship in the samples. The samples were kept at -70°C at the National Animal Disease Diagnostics and Epidemiology Centre (NADDEC), Entebbe, where the laboratory analysis was done.

### Sample processing and RNA extraction

One gram of tissue from each sample was weighed and homogenized using a homogenizer or a pestle and mortar with sterile glass. After homogenizing 9 ml of PBS was added and centrifuged in a refrigerated centrifuge at 10,000 rpm for 5 min to make 10% tissue suspension. The supernatant was decanted into a sterile tube and kept at 4°C for RNA extraction [25] and the pellet discarded into a disinfectant. The viral RNA extraction was carried out using QIAamp Viral RNA Mini kit following the manufacturer's guidelines. Extracted RNA was kept briefly at 4°C pending RT-PCR.

### Reverse Transcription and Polymerase chain reaction

Five microlitres extracted RNA was reverse transcribed to cDNA using Omniscript™ Reverse transcriptase kit (Qiagen, Germany). The reaction was carried out in 200 μl tubes with the following conditions: 1 μl of random hexamer, 0.125 mM each dNTPs, 10 units of ribonuclease inhibitor (MBI Fermentas), two units of Omniscript reverse transcriptase and the buffer provided by the manufacturer in a total volume of 20 μl. The reaction was carried out at 37°C for 60 min and stopped by incubation at 95°C for 10 min.

Three microliters of the reverse transcribed (cDNA) product was used as template for the PCR. The reaction was carried out in 200 μl thin walled PCR tubes, in a final volume of 25 μl containing the following reagents: 12.5 μl of DreamTaq PCR Master Mix (2×) containing ready to use Dream Taq DNA polymerase, buffer, MgCl_2 _and dNTPs (MBI Fermentas), 1 μl of forward and reverse primers (10 pmoles of each primer). The fusion gene specific primers used were (F1(F) 5'ATC ACA GTG TTA AAG CCT GTA GAG G 3' F gene 777-801 and F2(R) 5'GAG ACT GAG TTT GTG ACC TAC AAG C 3' F gene 1124-1148) [9,10]. PCR amplification was carried out as follows: Initial denaturation step 95°C for 5 min followed by denaturation at 94°C for 30 min, annealing at 50°C at 60 sec, extension at 72°C for 2 min for 35 cycles, and final extension at 72°C for 25 min. The length of the products was 372 bp [26].

### Analysis of PCR amplified products

The PCR amplicons were resolved on 2.0% agarose in Tris-borate- EDTA (TBE) buffer gels stained with ethidium bromide. Ten μl of the PCR product from each of the tubes were mixed with 1 μl of 6× buffer and electrophoresed along with 50 bp DNA molecular weight marker (GeneRular, MBI Fermentas) at a constant 80 V for 45 min in 1× TAE buffer. Amplified product was viewed under UV light and documented by a UV-transilluminator.

### Cloning and sequence analysis

PCR amplicons of the F gene were purified using QIAquick PCR Purification kit and sent over to Inqaba Biotec (South Africa) for sequencing. The sequencing involved cloning of ten representative RT-PCR products into a recombinant plasmid to obtain templates for sequencing. The recombinant plasmid carrying the insert from the representative clone was then subjected to automated DNA sequencing on ABI PRISM 310 Genetic Analyzer (Applied Biosystems, USA) and BigDye Terminator v3.1 Cycle sequencing kit (Applied Bio systems, USA). The sequence data were analysed with Finch TV and sequence similarity searches were conducted using the basic length alignment search tool (BLAST) of the National Centre for Biotechnology Information (NCBI). Complete alignment of nucleotide sequences was performed using ClustalW and deduced amino acids determined by MegAlign software version 5 (DNAStar, Inc., Madison, WI, USA). The phylogeny was inferred using neighbour-joining method in MEGA version 4.1 [27] by aligning 322 bp lineage specific nucleotide segment of the F gene with other sequences available in the GenBank. The robustness of groupings was assessed by bootstrap resampling of 1000 replicates and the tree visualized with same program

## Abbreviations

PPR: Peste des Petits Ruminants; PPRV: Peste des Petits Ruminants Virus; cDNA: complementary DeoxyriboNucleic Acid; VNT: Virus Neutralization Test; CIEP: Counter ImmunoElectroPhoresis; AGID: Agar Gel ImmunoDiffusion; RT-PCR: Reverse Transcriptase - Polymerase Chain Reaction; ELISA: Enzyme Linked ImmunoSorbent Assay; CCPP: Caprine Contagious PleuroPneumonia; BTV: BlueTongue Virus; FMD: Foot and Mouth Disease; TCRV: Tissue Culture Rinderpest Vaccine; NVRI: National Veterinary Research Institute; OIE: Organization of International Epizootics; IATA: International Air Transport Association; NADDEC: National Disease Diagnostic and Epidemiology Centre; DNA: DeoxyriboNucleic Acid; RNA: RiboNucleic Acid; BLAST; NCBI: National Centre for Biotechnology Information; MEGA: Molecular Evolutionary Genetics Analysis.

## Authors' contributions

All authors (PDL, JE, FNM, CA and DS) contributed equally in the study design, laboratory work, and manuscript preparation. All authors read and approved the final manuscript.

## Acknowledgements

The Authors are thankful to the National Veterinary Research Institute (NVRI), Vom for allowing the use of samples for the study. We are also grateful to Dr A.G.Lamorde scholarship for enhancing the completion of the work. We are greatly indebted to the staff of the diagnostic Laboratory of NADDEC, Entebbe for their technical assistance.

## Supplementary Material

Additional file 1 **Appendix 1: Permission to use samples for the study**. Permission obtained from the Executive Director of the National Veterinary Research Institute, Vom, Nigeria for the use of samples. Click here for file

Additional file 2 **Appendix 2: Import permit**. Import permit to ship in PPR suspected samples to from Nigeria into Uganda. Click here for file
