# Supplementary figures and images for: Molecular characterization and phylogenetic study of peste des petits ruminants viruses from North central States of Nigeria
Source: BMC Vet Res. 2011 Jul 4;7:32. doi: 10.1186/1746-6148-7-32 (PMC3141404; doi:10.1186/1746-6148-7-32)

**Appendix1**. Import permit to ship in samples into Uganda


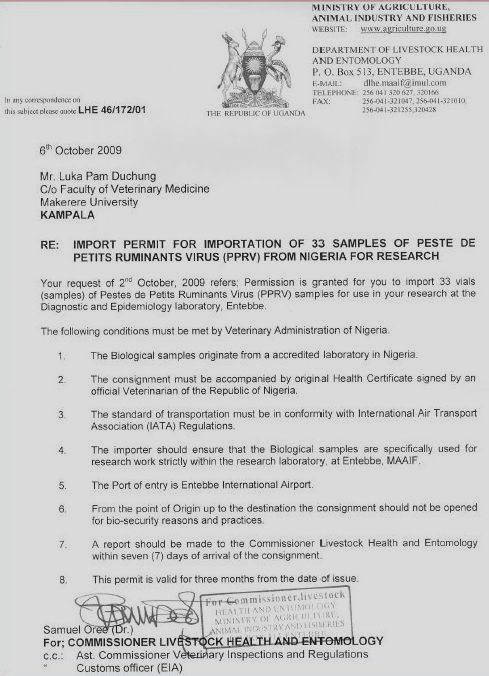

Supplement: Additional file 1 — Appendix 1: Permission to use samples for the study. Permission obtained from the Executive Director of the National Veterinary Research Institute, Vom, Nigeria for the use of samples. [file 1746-6148-7-32-S1.DOC]

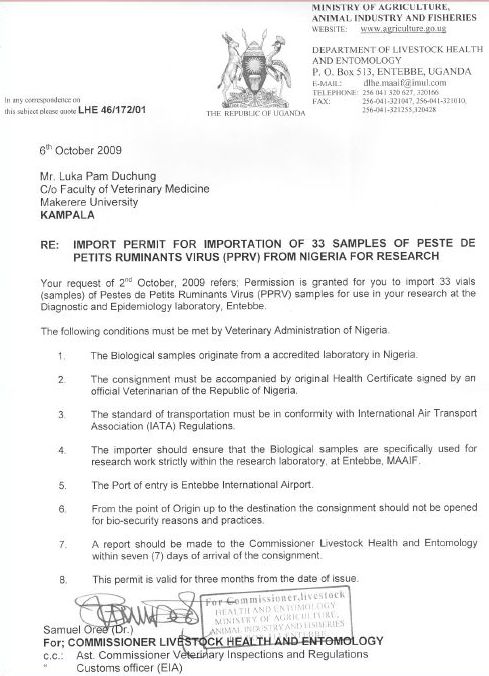

Supplement: Additional file 2 — Appendix 2: Import permit. Import permit to ship in PPR suspected samples to from Nigeria into Uganda. [file 1746-6148-7-32-S2.DOC]
